# An Inductor-Loaded Single-Port Planar Dual-Broadband Antenna with Stable Gains

**DOI:** 10.3390/mi14061233

**Published:** 2023-06-11

**Authors:** Wenxing An, Xinyu Xu, Jian Wang, Shenrong Li

**Affiliations:** 1 Tianjin Key Laboratory of Imaging and Sensing Microelectronic Technology, School of Microelectronics, Tianjin University, Tianjin 300072, China; 2 Qingdao Key Laboratory of Marine Information Perception and Transmission, Department of Ocean Information Engineering, Qingdao Institute of Ocean Engineering of Tianjin University, Qingdao 266200, China; 3 Tianjin Engineering Center of Integrated Circuit and Computing Systems, Tianjin 300072, China; 4 Wentai Technology Co., Ltd., Shanghai 200231, China; shenrong@wingtech.com

**Keywords:** base-station antenna, dual-band, inductive loading, wideband antennas

## Abstract

A single-port dual-wideband base-station antenna is reported here for mobile communication systems. Loop and stair-shaped structures with lumped inductors are adopted for dual-wideband operation. The low and high bands share the same radiation structure to accomplish a compact design. The operation principle of the proposed antenna is analyzed, and the effects of the lumped inductors are studied. The measured operation bands are from 0.64 GHz to 1 GHz and from 1.59 GHz to 2.82 GHz, with relative bandwidths of 43.9% and 55.8%, respectively. Broadside radiation patterns and stable gain with a variation of less than 2.2 dB are achieved for both bands. The inductor-loading technology is proven to be an effective way for dual-band antenna design with wide bandwidth and stable gain performance.

## 1. Introduction

Dual-band antennas have been widely used in modern wireless communication systems, as they have many favorable characteristics. Compared with single-frequency antennas, dual-band antennas can work on two or more frequency bands, avoiding the trouble of installing multiple antennas and saving installation space. They can decrease the cost of installing many single-frequency devices and reduce maintenance and management costs. A dual-band antenna can receive and transmit signals on multiple frequency bands, so they can improve the efficiency of signal reception and transmission, increase communication quality and speed, and enhance the system’s fault tolerance and anti-interference ability.

In recent years, base-station antennas have aroused widespread attention with the rapid development of 2G (Second Generation), 3G (Third Generation), 4G (Fourth Generation), and the coming 5G (Fifth Generation) wireless communication technologies. Many wideband antennas have been reported and investigated for base station applications. Instead of single-band performance, antennas with dual-band operation are much preferred because of the above advantages. For most mobile communication systems, it is popular for the frequency bands from 0.69 GHz to 0.96 GHz and from 1.71 GHz to 2.69 GHz to be covered simultaneously [1,2,3,4,5,6,7]. The dual-frequency antennas can be divided into single-port and dual-port. Single-port dual-frequency antennas have the advantages of simple structure, low cost, and easy installation. The dual-port dual-frequency antenna structure is more complex and has a high cost and a larger size compared with a single-port dual-frequency antenna.

There are already many dual-port dual-wideband antennas [8,9,10,11,12,13,14]. The proposed dual-band antennas with electromagnetic transparent elements have achieved a relative bandwidth of 14% at the higher band [8,9]. A dual-port multilayer stacked patch antenna was reported for dual-band operation [10]. The lower band is 22.9% from 764 MHz to 962 MHz, while the upper band is 20.3% from 1548 MHz to 1898 MHz. The shorting technology was introduced for wideband impedance matching. A dual-band dual-port antenna was proposed in [11] with a U-shaped PIFA (Planar Inverted F-shaped Antenna) for the lower band from 881 MHz to 913 MHz and an elliptical dipole for the upper band from 2.9 GHz to 5.35 GHz. The U-shaped PIFA surrounds the dipole so that both bands can share the same radiation aperture. In [12], a dual-band dual-port antenna was discussed for 2G, 3G, and 4G mobile communications. Different dipole elements are utilized for the low-frequency band from 800 MHz to 980 MHz and the high-frequency band from 1540 MHz to 2860 MHz. A shared-aperture antenna design was proposed for mobile communications [13,14] with the lower-band and upper-band elements at different heights. Although the above works have achieved satisfactory performance, these designs have employed independent port excitation for the lower- and upper-frequency bands, and this would make the system structure complicated and occupy more space.

Some single-port dual-band antennas can further reduce structural complexity and cost. A compact planar-printed monopole antenna was reported with two resonance modes [15]. The −10-dB impedance bandwidths are 6.4% from 2.35 GHz to 2.51 GHz and 19.5% from 4.90 GHz to 6.00 GHz, so this dual-band design can accommodate three WLAN (Wireless Local Area Network) bands (from 2.4 GHz to 2.484 GHz, from 5.150 GHz to 5.35 GHz, and from 5.725 GHz to 5.875 GHz). A single-port dual-band planar antenna was investigated in [16] with a compact structure for WLAN applications. L-shaped and E-shaped structures have been adopted for dual-band operation. A single-port dual-band asymmetric dipole antenna was designed for the laptop computer [17]. The meandered feed line with a rectangular patch works at 2.4 GHz and 5.2 GHz. Its asymmetric C-shaped parasitic part is for the 5.8 GHz band. A low-cost slot antenna was designed for a WLAN bands’ notebook metal cover [18]. A planar inverted F-shaped antenna (PIFA) with shorting strips was presented for the 2.4-GHz and 5.2-GHz WLAN [19]. The test −10-dB relative bandwidths are 5.8% (2.36 to 2.5 GHz) and 69% (2.94 GHz to 5.82 GHz). A dual-band monopole was proposed in [20] with shorting post and parasitic structures. The lower band is 10.4% from 2.37 GHz to 2.63 GHz, while the upper band is 14.3% from 5.52 GHz to 6.37 GHz. A dual-band antenna with a single port was explored in [21]. It acts as a microstrip antenna at the high-frequency band (4.8–6.9 GHz), while the coupling slot works independently at 2.4 GHz. A dual-band base-station filtering antenna was proposed for the LTE (Long Term Evolution) bands (2.5–2.7 GHz, 3.3–3.8 GHz) [22]. With four rectangular slots etched at the side of a metallic square patch, a slot-loaded antenna was reported in [23] with the dual operation. The lower band is from 3.28 GHz to 3.71 GHz, and the high-frequency band is from 4.8 GHz to 5.18 GHz. A single-port monopole antenna with a twin-stepped patch was reported for WLAN applications [24]. The impedance bandwidths are from 2.32 GHz to 3.03 GHz and from 4.77 GHz to 6.33 GHz, respectively. Etching T-shaped slots on the radiation structure, a dual-wideband crossed dipole was proposed for base-station applications [25]. The operation bands are from 1.67 GHz to 2.72 GHz and from 3.3 GHz to 3.9 GHz. With a loop and double-layer parasitic annular disk, a dual-band base station antenna was investigated for the 2G, 3G, 4G, and sub-6 GHz 5G systems [26]. The dual frequency relative bandwidths are 55.8% (1.55–2.75 GHz) and 14% (3.3–3.8 GHz). Magneto-electric dipoles were invented to achieve dual-wideband performance. In [27], a double-layer cross-magneto-electric dipole was reported for base-station communications with bandwidths of 51.3% for the lower band (1.68 GHz–2.84 GHz) and 11.4% for the upper band (5.31 GHz–5.95 GHz). A rectangle director was loaded above the magneto-electric dipole antenna in [28] with wide bandwidths of 46.7% (2.3–3.7 GHz) and 22.2% (5 GHz–6.25 GHz). A triple-layer magneto-electric dipole was studied in [29]. It can accommodate mobile communication bands, including 1.71–2.69 GHz and 3.3–5.0 GHz. Combining different magneto-electric dipoles, a dual-broadband dipole was designed and tested [30]. The bandwidths are 34% (0.78–1.1 GHz) and 49.5% (1.58–2.62 GHz). A dual-wideband dipole was presented with a double reflector [31]. Its relative bandwidths are 40.5% (0.68–1.03 GHz) and 50.6% (1.67–2.8 GHz). A triple-band dipole antenna was proposed for mobile communications [32]. The dipole has three different types of radiators covering three bands, including 0.69–0.96 GHz, 1.71–2.69 GHz, and 3.3–3.8 GHz. It has two reflectors and a relatively large height. The above works have been summarized in Table 1.

The above single-port antennas have realized dual-band characteristics with different structures. References [15,16,17,18,19,20,21] have single-layer designs, but their frequency bands are not wide enough for the mobile communication frequency bands of 33% (0.69–0.96 GHz) and 45% (1.71–2.69 GHz). To extend the bandwidth, multilayer structure designs were employed [22,23,24,25,26,27,28,29,30,31,32], and their impedance bandwidths have expanded significantly. Using double- and triple-layer structures, references [30,31,32] have accommodated the mobile communication bands from 0.69 GHz to 0.96 GHz and from 1.71 GHz to 2.69 GHz. However, the dual-broadband designs have a large gain variation of more than 3 dBi. Furthermore, the multilayer designs use only fundamental modes with different radiation structures for the low- and high-frequency bands. It would make the antenna structure relatively large and complicated with a high cost.

A planar single-layer structure with dual-wideband performance is desired for a compact, low-cost design. The high-order mode is inevitable for a single-layer design working at a high-frequency band. The reverse current would deteriorate the radiation performance, especially at the high frequency. It is necessary to control the reversed currents of high-order mode to achieve broadside radiation patterns and stable gain. As the inductor can provide a large reactance at high frequency and a small reactance at low frequency, it is possible to realize a dual-band performance utilizing its frequency-dependent reactance. There are already some antennas integrated with inductors. An inductor-loaded antenna was reported for GSM (Global System for Mobile)/DCS (Data Communication Subsystem) applications [33]. Using the inductively-loaded dipoles, a dual-band and wideband antenna with an omnidirectional radiation pattern was investigated for WLAN/UMTS (Universal Mobile Telecommunications System) applications [34]. However, their frequency bands need to be wider for wireless communication systems, so some novel designs with wider dual-operation bands are expected.

To fulfill the requirement for a compact structure with stable radiation performance, we designed a single-port planar dual-broadband antenna to accommodate the mobile communication bands. The first section briefs the related background of the dual-band antenna. Section 2 depicts the detailed structure of the single-port single-layer antenna. A planar single-layer single-port prototype was assembled, tested, and discussed in Section 3, to verify the dual-broadband design. Section 4 investigates the working principle of the dual-frequency antenna. The influence of the inductors on antenna performance is also analyzed. Section 5 provides some conclusions.

## 2. Materials and Methods

**Figure 1:**
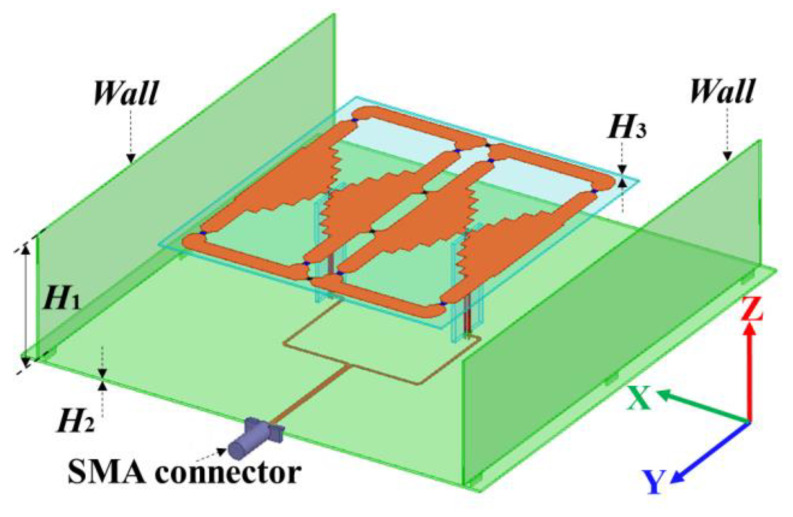
The perspective view of the proposed dual-band wideband antenna.

This dual-wideband antenna structure is shown in Figure 1. It consists of the top radiation structure, the bottom ground floor, the vertical feeding strips, and the side walls. The FR4 (Epoxy glass fiber) substrate has a relative dielectric constant of 4.1 and a loss tangent of 0.016. Single-layer radiation structures with single-port excitation are for a compact and low-cost design. Two vertical walls were placed in the polarization direction to improve its radiation performance. The detailed parameters are in Table 2.

**Figure 2:**
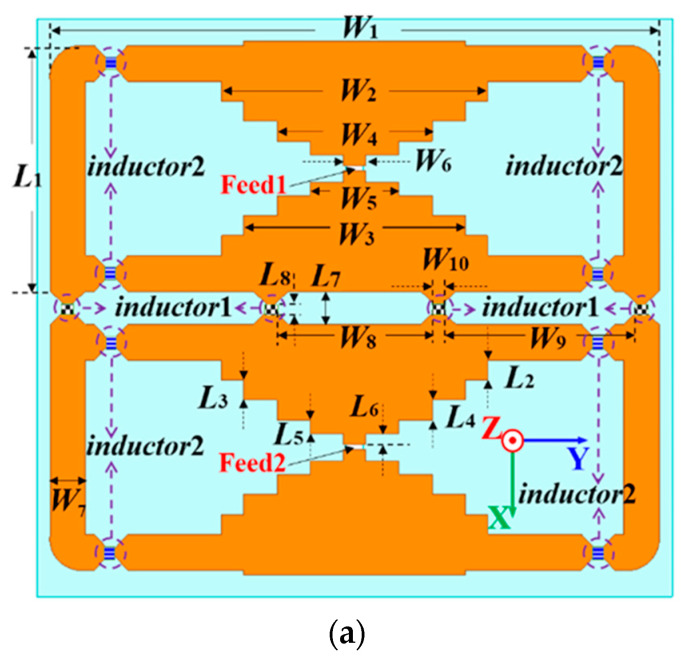

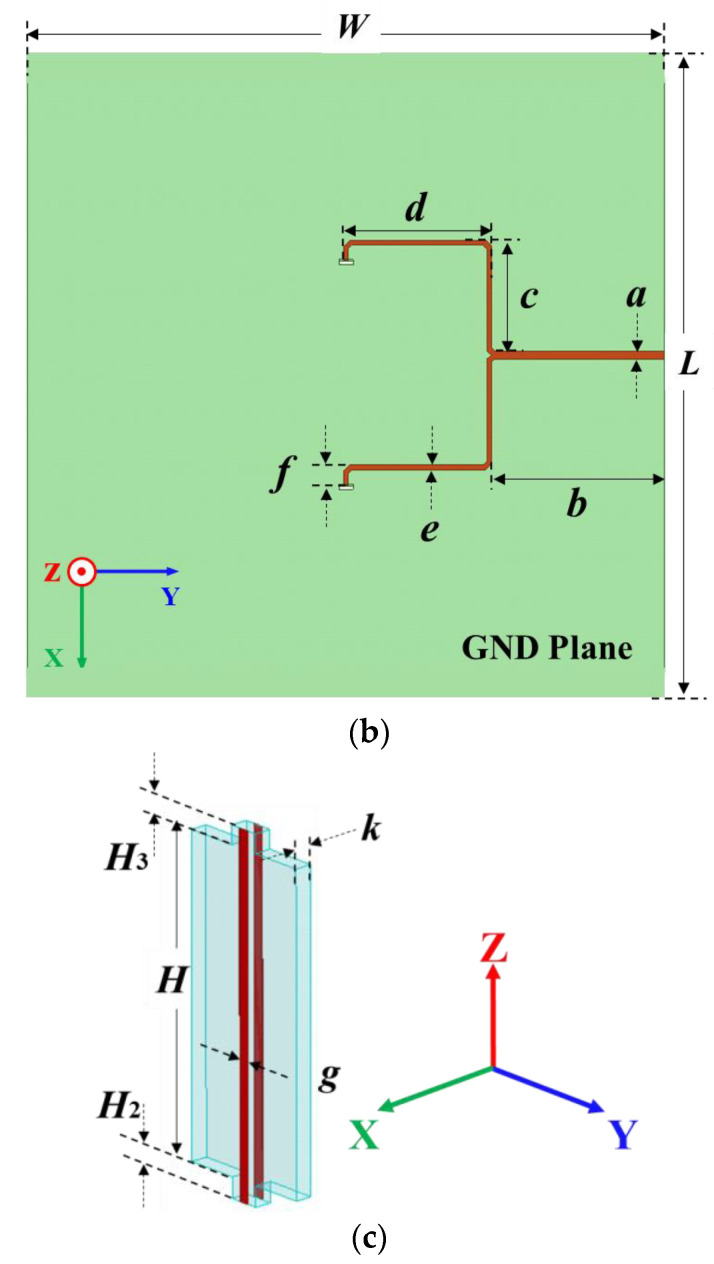
The single-port single-layer antenna: (**a**) top layer; (**b**) bottom layer; (**c**) vertical structure.

The single-layer structure is printed on the top FR4 substrate with a thickness of H_3_, as shown in Figure 2a. Two different kinds of inductors are employed for the antenna design. Inductors of type 1 and 2 have inductances of 8.2 nH and 6.8 nH, respectively [35,36]. The planar radiation structure with inductors is above the ground with a height of H+H_3_. The ground plane has the length and width of L and W, respectively. Two stair-cased structures are adopted for radiation. The stair-cased structure allows for a broadband characteristic. It can reduce the reflection coefficient and improve its impedance matching, leading to increased bandwidth. By properly designing the stair-cased gradient structure, it is possible to achieve a wideband performance. Two side arms with the length of L_1_ and width of W_7_ connect the stair-cased patch by inductors of type 2 to form a loop. There are two small loops on the top substrate. Four inductors of type 1 connect two small loops. The influences of these inductors will be discussed later. The feeding strips are at the center of two staircase-shaped structures for wideband excitation.

A dual-feed structure is designed for the antenna excitation, shown in Figure 2b,c. It includes the parallel transmission line on both sides of the vertical substrates, the power divider, and the microstrip transmission line on the bottom substrate. There are two pairs of vertical parallel feeding strips for the antenna excitation. The detailed vertical structure is shown in Figure 2c. The tops of the vertical parallel transmission line are connected to the staircase-shaped structure. The bottoms of the parallel transmission line are soldered to the ground and the feeding strip, respectively. A power divider is introduced into the bottom-feeding structure to realize an in-phase excitation for the two loop structures. The SMA connector is soldered at the end of the microstrip transmission line for the excitation of both bands.

The parallel line feeding structure has a wide operating bandwidth. It can transmit signals over a range of frequencies without significant distortion. The compact design is easy to integrate with microwave circuits, such as filters, couplers, and baluns. These favorable features make it popular for microwave components, small devices, and systems.

## 3. Results

**Figure 3:**
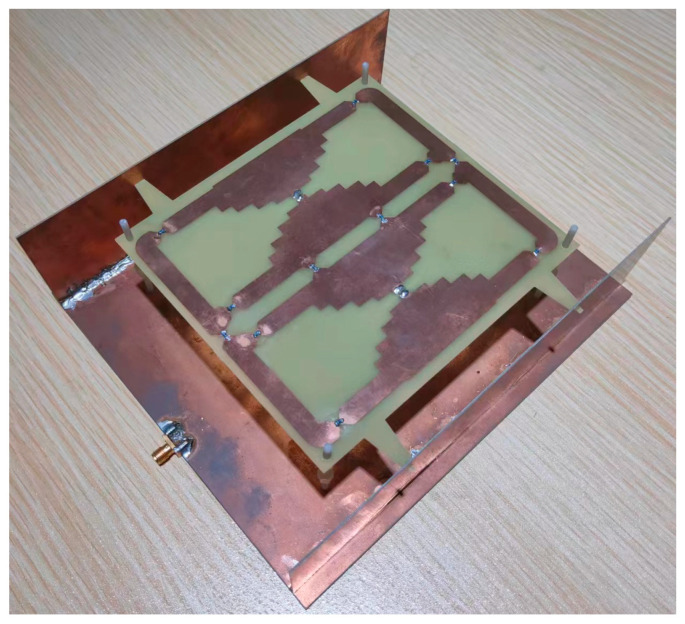
Dual-band antenna prototype.

**Figure 4:**
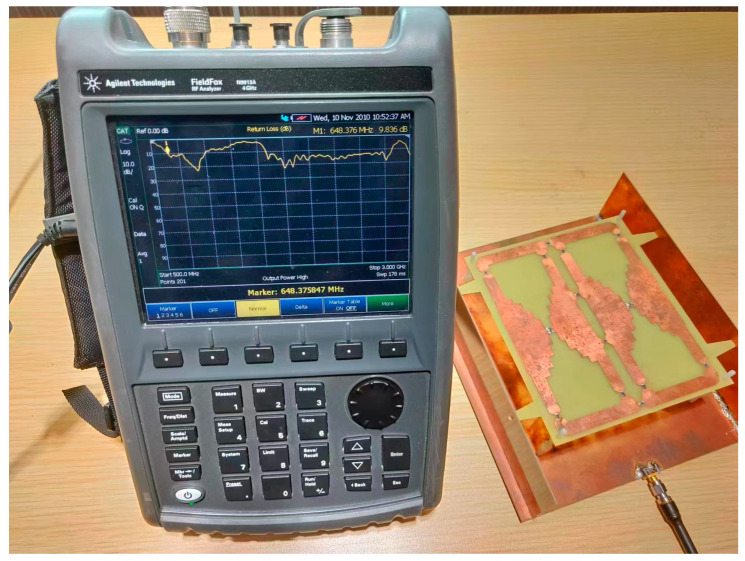
Testing scenario with Agilent N9913A network analyzer.

**Figure 5:**
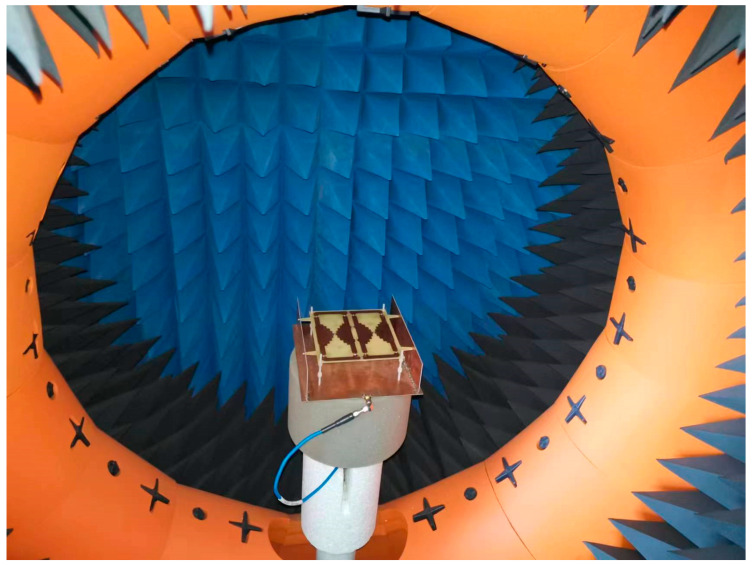
Testing scenario with SATIMO StarLab.

The prototype of a single-port single-layer antenna loaded with inductance was processed and manufactured to verify the proposed design, as shown in Figure 3. The top, bottom, and vertical structures were fabricated using PCB (Printed Circuit Board) technology. Surface-packaged inductors are on the top surface. The antenna structure is supported by Teflon plastic bolts fixed on the ground plane. The Agilent N9913A network analyzer and SATIMO StarLab spherical near-field test system were used for the performance measurement. The test scenarios are shown in Figure 4 and Figure 5. The equipment parameters of the Agilent N9913A network analyzer and SATIMO StarLab are shown in Table 3 and Table 4. The dual-band model was calculated with the commercial software High-Frequency Structure Simulator (HFSS). HFSS is a 3D electromagnetic simulation software to design and analyze high-frequency electronic devices. The software uses the finite element analysis method to solve Maxwell’s equations for electromagnetic fields in complex geometries such as antennas, microwave circuits, RF modules, and electromagnetic interference (EMI) and electromagnetic compatibility (EMC) problems. It is applicable to aerospace, defense, telecommunications, and electronics.

The spherical near-field test system is a technique used to evaluate the radiation performance by measuring the near-field electromagnetic field of the antenna to be tested. This SATIMO StarLab spherical near-field test system has fifteen probes and a rotating platform. The antenna under testing is on the central platform of the spherical measurement system. During the testing process, the platform rotates 360 degrees. Meanwhile, fifteen probes measure the electromagnetic field of the antenna under test at various angles. The measured data taken by the probe was utilized to calculate the far-field radiation pattern of the antenna. It uses the Fourier transform to process the near-field measurement data. Furthermore, the spherical near-field test system has the advantages of compact size and low cost over other far-field antenna testing systems.

**Figure 6:**
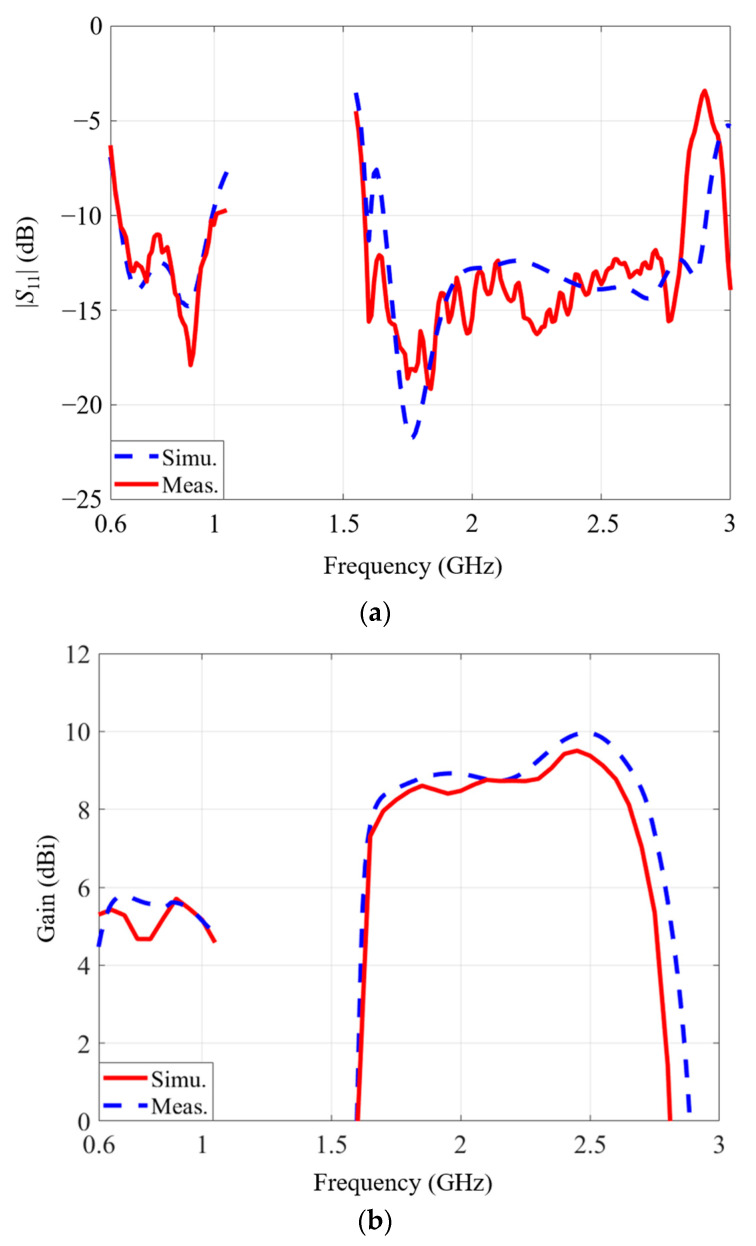
Tested and calculated antenna performances: (**a**) S-parameter, (**b**) gain.

**Figure 7:**
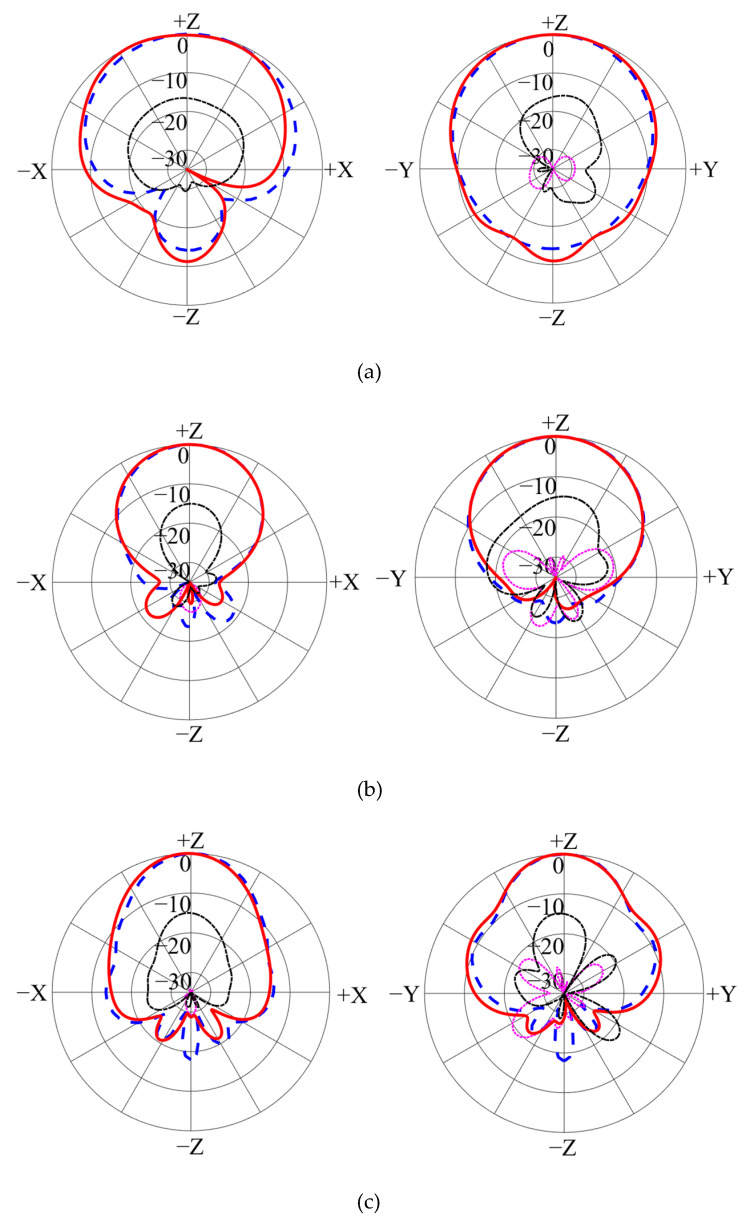
Calculated and tested radiation patterns: (**a**) 0.8 GHz; (**b**) 1.8 GHz; (**c**) 2.6 GHz.

The calculated and tested results are depicted in Figure 6 and Figure 7. The simulated and tested curves agree well with each other. The calculated −10-dB frequency band is from 0.63 GHz to 1.01 GHz. The test −10-dB bandwidth is from 0.64 GHz to 1 GHz. For the higher band, the calculated -10-dB band is from 1.7 GHz to 2.93 GHz, while the measured −10-dB band is from 1.59 GHz to 2.82 GHz. Stable radiation performance is realized within the target band. For the lower band (0.69–0.96 GHz), the tested gain fluctuates between 4.7 dBi and 5.7 dBi. The average value is 5.2 dBi. For the higher band (1.71–2.69 GHz), the fluctuation range of the tested gain is from 7.3 dBi to 9.5 dBi, and the average value is 8.6 dBi.

The radiation patterns are plotted in Figure 7. It is clear that broadside radiation patterns are realized for both bands, and the measured co-polarization results almost overlap with the simulation ones. The front-to-back ratio at 800 MHz is more than 10 dB, and the cross-polarization levels are less than −12 dB. At high frequencies, the back radiation levels are at least 20 dB lower than the frontside radiation. The cross-polarization levels are at least 14 dB lower than the co-polarization level.

Certain discrepancies exist between the calculated and tested results, especially at the higher band near 2.7 GHz. One possible reason is the intrinsically unstable inductance of the adopted inductors. The datasheets [35,36] indicate that the inductance of both types increases by about 10% from 2 GHz to 3 GHz. The large inductance would lead to a relatively large electrical length. It would make the frequency band slightly shift to a lower frequency. This can explain the deviations between the simulation of measurement results between 2.5 GHz and 3 GHz. Another reason for this is the assembly error. For this specific design, lumped inductors were soldered manually to the metallic structure. Therefore, it is difficult to assure that every lumped inductor is soldered uniformly, so some phase errors would exist, which would cause relatively higher measured cross-polarization levels.

## 4. Discussion

To further understand this dual-band antenna, the influence of two kinds of inductors on the antenna performance was analyzed. Then, the current distributions of the top metallic structure were analyzed to clarify its working mechanism.

**Figure 8:**
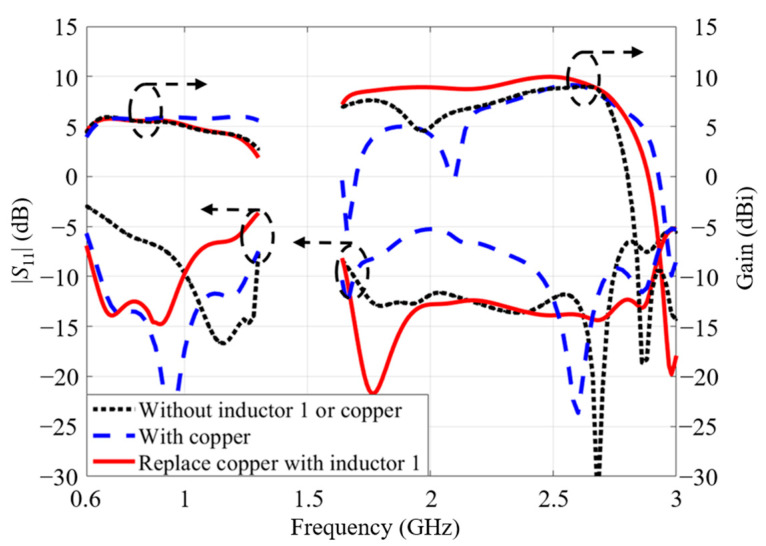
The influence of inductor 1 on the antenna performance.

Firstly, the effect of inductor 1 was analyzed. Meanwhile, inductor 2 remained unchanged. As inductors of type 1 connect two small loop structures, the antenna performances are analyzed in three cases. In case 1, there was no connection between the two small loops. In case 2, copper strips connected two small loop structures. In case 3, inductors of type 1 connected two loops. The S-parameters and gains of these three cases are plotted in Figure 8. The black dot curves represent the antenna performance of case 1. When two loops are disconnected, the antenna has a reduced size. Although the antenna can maintain its dual-band performance, the -10-dB band at a low frequency shifts to a high frequency; it is from 1 GHz to 1.3 GHz. Therefore, the antenna of case 1 cannot cover the low-frequency band between 0.69 GHz and 0.96 GHz. To accommodate the lower target band, two small loops were connected by copper strips to increase the effective length. This can be seen as the blue dash curves for case 2. However, the impedance matching at higher bands deteriorates. The antenna gain has a variation. The inductors are loaded to connect two loops instead of copper to improve the impedance matching. It is observed in Figure 8 that dual-wideband performance is achieved with stable gains when inductors of type 1 are loaded. Therefore, inductors of type 1 can realize better impedance matching and stable radiation performance, especially for the upper band.

**Figure 9:**
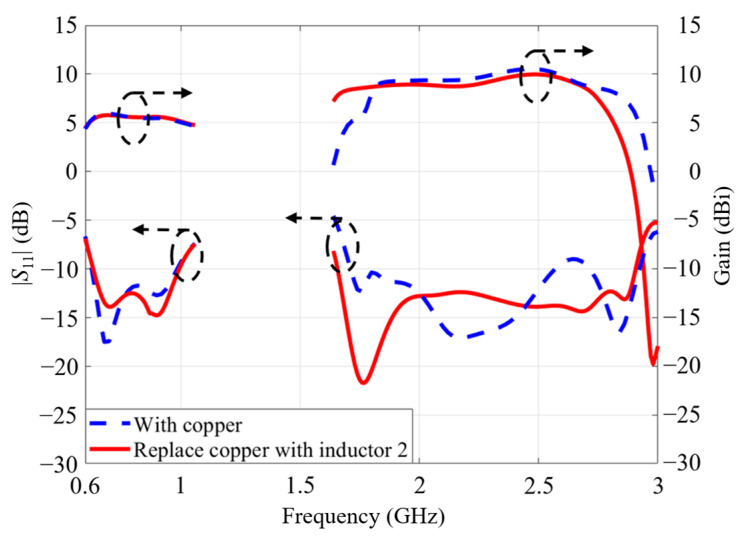
The influence of inductor 2 on the antenna performance.

Secondly, the influence of inductor 2 was analyzed. Inductors of type 1 remained unchanged. As inductors of type 2 unite the stair-cased structure and the side arms, the antenna performance was compared with that united by the copper strips. The antenna performances are plotted in Figure 9.

The blue dash curves represent the antenna performance with the copper strip, while the red solid curves are with the antenna performance with inductor 2. It was noticed that inductor 2 had almost no effect on the low-frequency performance. The antenna gain and S-parameter were relatively stable regardless of the inductance or copper strip connection. The reason for this is that the inductor 2 has a relatively small inductance at a low frequency. For the high-frequency band, the antenna gain with the copper strip drops dramatically at 1.7 GHz. Inductor 2 was adopted to realize stable gains at a higher band, because it can provide a larger inductance and increase the effective length, especially at high frequencies. It is clear that the −10-dB band and gain slightly move to the low frequency after replacing the copper strip with inductor 2.

**Figure 10:**
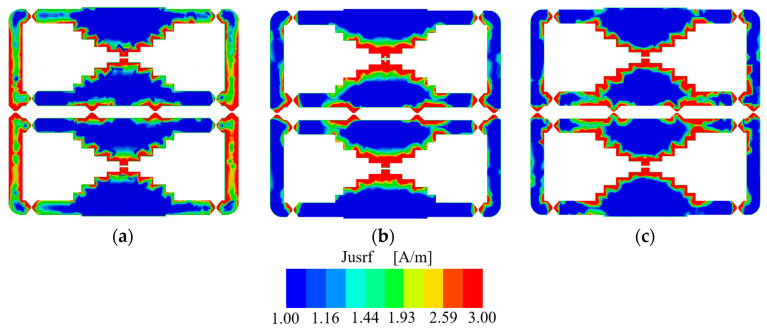
Effective current distributions of the dual-band antenna: (**a**) 0.8 GHz; (**b**) 1.9 GHz; (**c**) 2.6 GHz.

**Figure 11:**
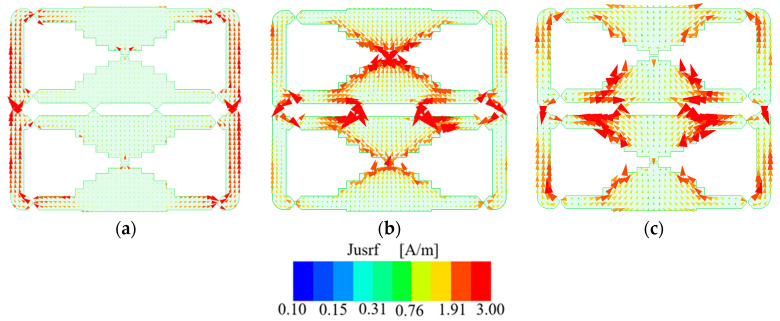
Instantaneous current distributions of the dual-band antenna: (**a**) 0.8 GHz; (**b**) 1.9 GHz; (**c**) 2.6 GHz.

The effective and instantaneous current distributions are plotted in Figure 10 and Figure 11 at 0.8 GHz, 1.9 GHz, and 2.6 GHz to investigate its working mechanism. It is observed from Figure 10 that there are current distributions at the stair-cased structure and side arms. The effective current distributions have the minimum points at the bottom and top stair-cased shapes. However, the effective current distributions in the high-frequency and low-frequency bands are different. There are minimum current points at the side arms at the high-frequency band. No minimum points exist at the side arm at the low frequency. Therefore, this antenna operates at different modes in the low-frequency and high-frequency bands.

It is observed from Figure 11 that the current directions are different between the low- and high-frequency bands. At low frequencies, the side-arm currents maintain in the same direction. The antenna is with the one-wavelength mode of the whole loop structure. At high frequencies, most currents flow in the same direction. However, reverse currents exist near four inductors of type 1 that connect two small loops. Therefore, high order mode is excited at a high frequency. Because the reverse currents occupy a small area, they have limited influence on the radiation patterns.

Based on the above analysis, this dual-band antenna works in the fundamental mode at the low frequency and high order mode at the high frequency. Inductors of type 1 connect two small loops, which are mainly responsible for the low-frequency band. The inductor 2 is helpful for the high-frequency target band.

## 5. Conclusions

A single-port dual-wideband base antenna can be used for the sub-3-GHz wireless communication bands. Based on the planar single-layer structure, fundamental and high-order modes have been stimulated for the low- and high-frequency bands, respectively. With the introduced lumped inductors, the dual-broad band performance was realized. This antenna can cover the sub-3-GHz frequency bands (0.69–0.96 GHz and 1.71–2.69 GHz). Satisfactory radiation performances are achieved with stable gains and low back-lobe levels. The proposed inductor-loading technology can utilize the fundamental and high-order modes for dual-wideband and stable performance. It can enlighten more advances for future antenna designs.

## Figures and Tables

**Table 1:** Comparison of this design with published dual-band works.

Ref.	Low Band High Band (GHz)	Relative Bandwidth (%)	Gain Variation (LB, HB) (dB)	Radiation Structure Layers	Single Port for Dual-Band
[8]	1.65~2.7, 3.3~3.8	48.3%, 14%	2, 1.4	2	No
[9]	1.8~2.7, 3.3~3.8	40.0%, 14%	2, 3	2	No
[10]	0.764~0.962, 1.548~1.898	22.9%, 20.3%	N.A.	2	No
[11]	0.88~0.91, 2.9~5.35	3.6%, 59.5%	0.4, 2.1	1	No
[12]	0.78~0.98, 1.47~3	22%, 68%	1, 0.8	1	No
[13]	0.69~0.96, 1.7~2.7	32.7%, 45.5%	1.2, 1.4	2	No
[14]	0.69~0.98, 1.7~2.73	36%, 46.5%	0.3, 1.7	4	No
[15]	2.35~2.51, 4.90~6	6.4%, 19.5%	0.6, 0.4	1	Yes
[16]	2.39~2.5, 5~6.06	4.5%, 19.2%	0.1, 2.5	1	Yes
[17]	2.37~2.56, 5.06~5.39	7.7%, 6.3%	0.3, 0.5	1	Yes
[18]	2.4~2.5, 4.75~6	4.1%, 23.3%	0.2, 1	1	Yes
[19]	2.36~2.5, 2.94~5.8	5.8%, 69%	0.2, 2.5	1	Yes
[20]	2.37~2.63, 5.52~6.37	10.4%, 14.3%	3, 2.5	1	Yes
[21]	2.27~2.5, 4.8~6.9	9.6%, 35.9%	4, 3.5	1	Yes
[22]	2.5~2.7, 3.3~3.8	7.7%; 14%	1.5, 1	3	Yes
[23]	3.28~3.71, 4.8~5.18	12.3%, 7.6%	0.78, 2.16	2	Yes
[24]	2.32~3.03, 4.77~6.33	26.5%, 28.1%	N.A.	2	Yes
[25]	1.67~2.72, 3.3~3.9	47.8%, 16.6%	1.2, 0.9	2	Yes
[26]	1.55~2.75, 3.3~3.8	55.8%, 14%	0.7, 0.4	3	Yes
[27]	1.68~2.84, 5.3~5.95	51.3%, 11.4%	3.5, 2.5	2	Yes
[28]	2.3~3.7, 5~6.25	46.7%, 22.2%	0.7, 3.2	3	Yes
[29]	1.74~2.6, 3.34~5.08	39.6%, 41.3%	1.1, 6.2	3	Yes
[30]	0.78~1.1, 1.58~2.62	34%, 49.5%	1.5, 4	2	Yes
[31]	0.68~1.03, 1.67~2.8	40.5%, 50.6%	1, 3.5	2	Yes
[32]	0.7~0.96, 1.7~3, 3.3~3.8	31.3%, 55.3%, 14%	2, 3, 0.9	2	Yes
This work	0.64~1, 1.59~2.82	43.9%, 55.8%	1, 2.2	1	Yes

**Table 2:** Antenna parameters of the simulation model (mm).

Para.	*L*	*L*1	*L*2	*L*3	*L*4	*L*5	*L*6	*L*7
Value	181	55.25	4.5	4.5	4.5	3	2.54	7.5
Para.	*L*8	*W*	*W*1	*W*2	*W*3	*W*4	*W*5	*W*6
Value	1	205	137.5	60	50	35	20	5
Para.	*W*7	*W*8	*W*9	*W*10	*H*	*H*1	*H*2	*H*3
Value	8	36.54	44.54	1.3	43.4	55	0.9	0.7
Para.	*a*	*b*	*c*	*d*	*e*	*f*	*g*	*k*
Value	2	57.13	30.75	45.75	0.75	5.72	0.75	1.1

**Table 3:** The parameter of the Agilent N9913A network analyzer.

Parameter	Performance
Frequency range	30 KHz~4 GHz
Port	2
Dynamic range	>100 dB
Amplitude accuracy	±0.5 dB
Temperature range	−10~+55 °C

**Table 4:** The parameter of the SATIMO StarLab.

Parameter	Performance
Frequency range	650 MHz~6 GHz
Typical dynamic range	70 dB
Peak gain accuracy	>±1.5 dB
−10 dB sidelobe accuracy	>±1.6 dB
−20 dB sidelobe accuracy	>±4.6 dB

## Data Availability

Not applicable.
